# Focused ultrasound therapy for Alzheimer’s disease: exploring the potential for targeted amyloid disaggregation

**DOI:** 10.3389/fneur.2024.1426075

**Published:** 2024-08-06

**Authors:** Kurt Scott, Stephen P. Klaus

**Affiliations:** ^1^TCD School of Medicine, Trinity College Dublin, Dublin, Ireland; ^2^Department of Neurology, St. James’s Hospital, Dublin, Ireland

**Keywords:** focused ultrasound, Alzheimer’s disease, microbubble, blood-brain barrier, amyloid-beta disaggregation

## Abstract

**Introduction:**

Alzheimer’s disease, a progressive neurodegenerative disorder, is marked by beta-amyloid plaque accumulation and cognitive decline. The limited efficacy and significant side effects of anti-amyloid monoclonal antibody therapies have prompted exploration into innovative treatments like focused ultrasound therapy. Focused ultrasound shows promise as a non-invasive technique for disrupting the blood–brain barrier, potentially enhancing drug delivery directly to the brain and improving the penetration of existing therapeutic agents.

**Methods:**

This systematic review was conducted using PubMed and Embase databases, focusing on studies published in the last ten years that examined the use of low–intensity focused ultrasound for blood–brain barrier disruption in Alzheimer’s disease. The search strategy encompassed terms related to Alzheimer’s disease, focused ultrasound, and the blood–brain barrier. Studies were selected based on predefined inclusion and exclusion criteria. The quality of included studies was assessed using the Oxford Centre for Evidence-Based Medicine Levels of Evidence framework.

**Results:**

Twelve studies were analyzed, the results of which suggested that low intensity focused ultrasound when combined with microbubbles may safely and transiently disrupt the blood–brain barrier. These studies, primarily early-phase and observational, highlight the potential feasibility of focused ultrasound in facilitating drug delivery to the brain for the treatment of Alzheimer’s disease. Notably, one study reported positive impacts on cognitive tests, suggesting potential direct therapeutic effects of focused ultrasound beyond blood–brain barrier disruption.

**Conclusion:**

The results of the included studies indicate the use of focused ultrasound in Alzheimer’s disease treatment might be safe and effective in transiently opening the blood–brain barrier. Although current evidence is promising, further research is needed to establish generalizability. Future studies should also aim to further elucidate the mechanisms of action of low-intensity focused ultrasound as well as microbubbles for blood–brain barrier opening and explore potential clinical benefits beyond blood–brain barrier opening such as impacts on cognitive outcomes. Future studies should also aim for greater participant diversity to ensure findings are applicable across the full spectrum of Alzheimer’s disease patients.

## Introduction

Alzheimer’s disease (AD) is a progressive neurodegenerative disorder characterized by the accumulation of beta-amyloid plaques in the brain as well as cognitive decline (1). Despite offering a glimmer of hope as a treatment for AD, anti-amyloid monoclonal antibody therapies have shown limited efficacy and significant side effects (2), which has prompted the exploration of other innovative therapy modalities such as focused ultrasound therapy (FUS). FUS was successfully used to disrupt the blood-brain barrier (BBB) in several animal studies (3). Having emerged through preclinical trials, this treatment modality is now progressing to a limited number of clinical trials, as a potential non-invasive approach for disrupting the BBB. BBB opening might enable the use of therapeutic agents, for the treatment of AD, that would otherwise be unable to reach targets in the brain (4). Additionally, it might improve the penetration of therapeutic agents currently used to treat AD (5). This systematic review aims to evaluate the potential application of FUS to disrupt the BBB in AD, thereby illuminating its potential role in the treatment of AD in the future.

## Methods

### Search strategy

The search was conducted on the 12^th^ of March 2024 using PubMed and Embase. For each database the search encompassed terms related to AD, FUS, and the BBB. Detailed search strategies were appropriately adapted for each database, using suitable keywords, MeSH terms, Boolean operators, and filters. The search strings used are available on request. All studies identified were independently screened by both investigators. Additionally, references to relevant articles were also manually searched to identify suitable studies not yielded by the initial searches. The results of this systematic review were recorded according to PRISMA guidelines (6).

### Inclusion and exclusion criteria

#### Inclusion criteria

- Studies available in English.
- Studies that are peer reviewed.
- Human studies.
- Studies focusing on the use of focused ultrasound for blood-brain barrier opening.
- Studies involving participants diagnosed with Alzheimer’s disease.
- Studies available in abstract and full-text format.
- Clinical trials, multicenter studies, pilot studies, controlled clinical trials, RCTs, meta-analyses, observational studies.
- Published in the last 10 years.

#### Exclusion criteria

- Studies not available in English.
- Studies that are not peer reviewed.
- Animal studies.
- Studies not focusing on the use of focused ultrasound for blood-brain barrier opening.
- Studies not involving participants diagnosed with Alzheimer’s disease.
- Studies not available in abstract or full-text format.
- Published more than 10 years ago.
- Review articles, opinion pieces, editorials, systematic reviews, literature reviews.

### Quality assessment

We employed the Oxford Centre for Evidence-Based Medicine (OCEBM) Levels of Evidence framework (7) to assess the quality of the studies included in this systematic review. This framework categorizes studies based on their methodological design, providing a structured approach to evaluate the potential for bias and the strength of evidence offered. Each study was critically appraised and assigned a level from 1 to 5, with level 1 representing the highest quality evidence for example RCTs with a very low risk of bias and level 5 representing lower quality evidence such as expert opinion. This grading made it possible to contextualize the findings of each study within the spectrum of evidence quality, therefore ensuring a more comprehensive synthesis of the data yielded by the search and screening process. The application of the OCEBM Levels of Evidence framework facilitated a clear and systematic assessment of the likely reliability of each study and its respective contribution to our understanding the therapeutic potential of FUS.

### Data extraction and analysis

The results of the PubMed and Embase searches as well as the two studies identified from other sources were compiled in Excel. Deduplication involved the use of automatic Excel functions as well as manual screening by both researchers. Following this, a two-stage screening process was implemented: (1) abstract screening to exclude studies based on inclusion and exclusion criteria, (2) full-text review to refine the selection to 12 studies which fully satisfy the inclusion and exclusion criteria. The two-stage screening process was done by both researchers. There were no disagreements following the screening process therefore third-party mediation wasn’t necessary. The following details were extracted from the 12 included studies: author, year of publication, participant age and sex, intervention, and outcomes. Thematic analysis was employed by both researchers to identify themes and trends in the extracted data.

## Results

### Study selection

**Figure 1:**
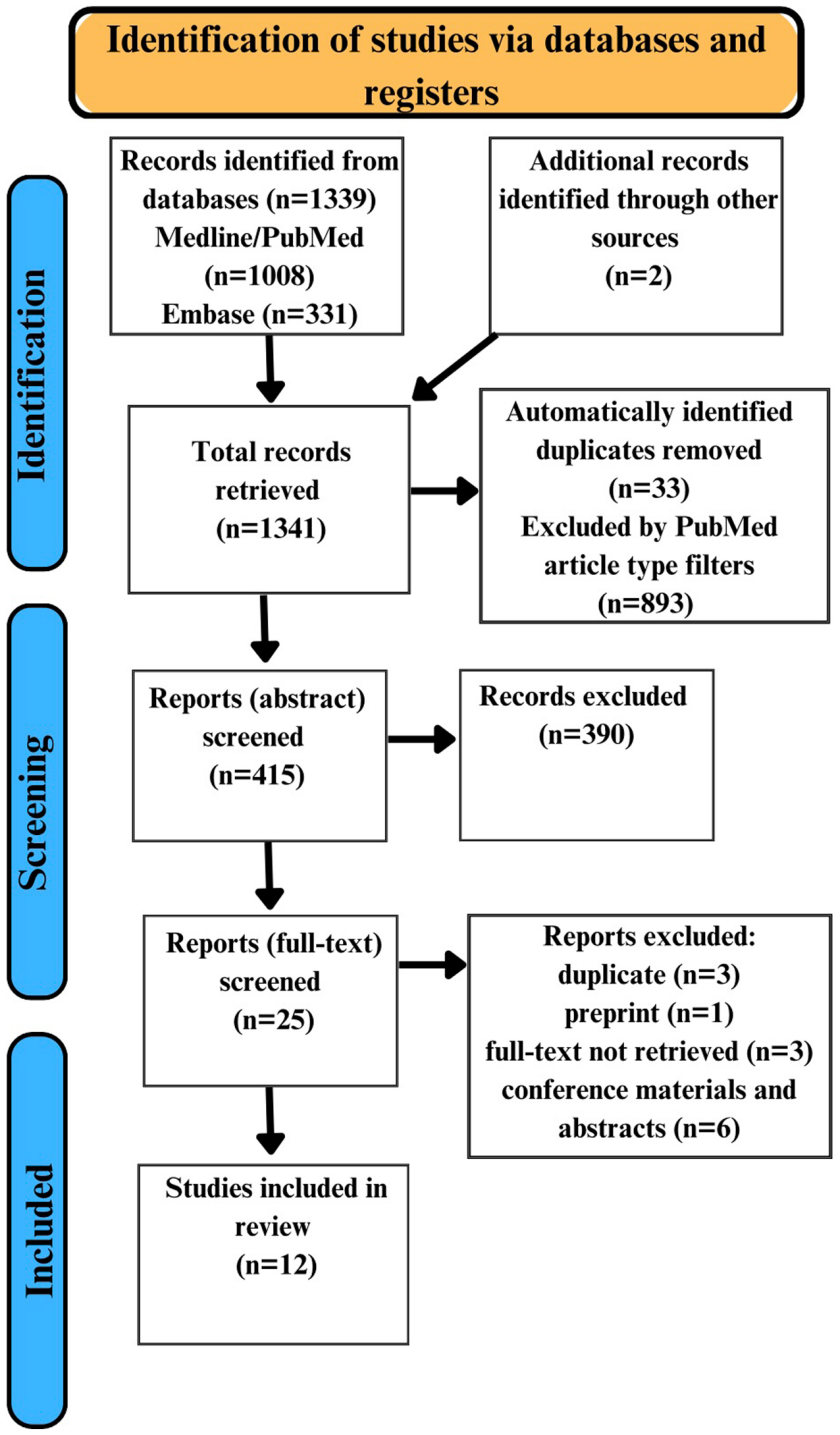
Study Selection Flow Diagram.

Overall, our search strategy yielded 1,339 articles. Additionally, 2 articles were identified by reference list searching. Of the 1,341 yielded articles, 33 duplicates were automatically identified, and 893 articles were automatically excluded using PubMed filters based on article type. 415 articles were manually screened for duplicates and assessed for eligibility through abstract screening. We excluded 390 articles based on the abstracts screening. Based on a full-text review of 25 articles another 13 articles were excluded for various reasons. 12 studies were identified as fully satisfying the inclusion and exclusion criteria (Figure 1).

### Characteristics of the included studies

This systematic review comprehensively analyzed 12 studies investigating the use of low intensity FUS to facilitate BBB disruption in patients with AD. The included studies were all conducted between 2018 and 2023. 10 of the studies employed FUS in conjunction with systemic microbubbles, while 2 studies employed FUS without microbubbles. These studies targeted various brain regions, including the hippocampus, frontal lobes, parietal lobes, and entorhinal cortex. The primary objective across these studies was to evaluate the safety, feasibility, and preliminary efficacy of FUS-mediated BBB disruption in AD. The 12 included studies encompass a range of design types: Phase II clinical trials, Phase I/II clinical trials, open-label prospective trials, and pilot studies. This reflects the early-phase exploration of FUS in this field. All included studies were assigned an OCEBM level of 4, underscoring the observational and early-phase nature of research into this topic. This level reflects the studies’ focus on safety, feasibility, and initial efficacy without the use of randomized control groups.

Sample sizes varied from a minimum of 3 participants (8) to a maximum of 35 participants (9). The age and sex of participants across all studies was reflective of the demographics of people most commonly affected by AD (10, 11); a majority of the studies reported mean participant ages of greater than 65 years and most of the studies report more female participants than male participants. Geographically, the studies spanned the United States (8, 12–15), Canada (16–18), France (19), Germany (9), and South Korea (20, 21), highlighting the global interest in exploring FUS for AD treatment. Funding sources included both industry and public institutions.

### Intervention methodology

All 12 of the included studies used low-intensity transcranial FUS for BBB disruption. The Insightec system, which is equipped with a helmet-like device containing multiple transducer elements that can be individually controlled and focused, was used in 6 out of the 12 studies (8, 12–14, 16, 18). SonoCloud-1, which is an implantable device, was used in one of the 12 studies (19). The Neurosona system, which specific details about were not provided in either study, was used in 2 out of the 12 studies (20, 21). The specific transcranial FUS system used to disrupt the BBB wasn’t mentioned in 3 out of the 12 studies (9, 15, 17). In all 12 studies target sites were sonicated sequentially rather than concurrently. While the specific rationale for sequential sonication is unclear, given the observational and early-phase nature of research into this treatment modality, this approach might have been chosen because sequential sonication simplifies the interpretation of results.

10 of the 12 studies used microbubbles (8, 12–14, 16–21). The most used microbubble agent was Definity, which was used in 9 out of the 10 studies. The microbubble agent SonoVue was used in the other study. In all studies where microbubbles were used, the route of administration was intravenous. This route of administration allows microbubbles to rapidly reach the blood vessels in the brain. Consequently, sonication was performed either simultaneously with (14, 16, 17, 19) or shortly following (8, 12, 13, 18, 20, 21) the administration of the microbubbles in all such studies. The treatment protocols varied significantly in terms of sonication duration, repetition time, and treatment frequency. For example, one study employed a single-session protocol (20), whereas another implemented a regimen of seven sessions over 3.5 months (19). The protocols and parameters used in each study are available in greater detail in the results table in Supplementary materials. Notably, the target areas differed across studies, ranging from specific regions like the hippocampus and entorhinal cortex to broader regions including the frontal and parietal lobes.

### Key outcomes

No serious adverse events related to the procedure were reported in any of the 12 studies. 1 of the 12 studies reported 1 severe adverse event during the trial, but it was concluded to be unrelated to the BBB opening procedure (19). In 8 of the 12 studies successful BBB opening immediately after sonication was reported, of which 7 studies reported successful BBB opening in 100% of sessions (8, 12–14, 16–18) and 1 study reported successful BBB opening in 62.5% of sessions (19). It’s important to note that BBB opening was not measured after all sessions in the studies. The methods used for confirmation of BBB opening and closure varied across the 12 studies. MRI imaging and gadolinium-based contrast agents were used in most of the studies. The imaging techniques used by each study is detailed further in the results table in Supplementary materials. 2 of the 12 studies did not achieve successful BBB opening in any session (20, 21). However, it was noted in both articles that BBB opening was likely not achieved due to sub-therapeutic acoustic pressure being used. 2 of the 12 studies did not specifically measure BBB opening (9, 15). All studies that reported successful BBB opening also reported subsequent apparent closure of the BBB within 48 h of sonication. No studies reported negative effects on cognitive measures in their follow up periods. However, it’s important to note that not all studies considered cognitive measures during their follow up periods and the follow up periods varied between studies. One study reported a positive impact on certain cognitive tests following FUS treatment. Specifically, they reported improvements in immediate recall and recognition memory on the Seoul Verbal Learning Test after the sonication procedure (20).

## Discussion

### FUS therapy for AD

The results of this systematic review contribute to the evolving narrative surrounding the application of FUS in neurodegenerative disorders, particularly AD. The results of the 12 included studies indicate that FUS with microbubbles may be able to safely and transiently disrupt the BBB. As research into this topic is in its early stages, as evidenced by all 12 studies being assigned OCEBM level 4, these results are not generalizable. However, successful transient BBB opening without severe adverse events reported in the 12 studies warrants further research into this topic. Further, the sonication parameters used in these studies will most likely help to inform the methodology of future trials on this topic. This BBB disruption may facilitate the delivery of certain therapeutic agents directly to the brain, potentially revolutionizing treatment paradigms for AD by overcoming a crucial obstacle in drug delivery (22).

### Microbubbles

In the process of opening the BBB with FUS, microbubbles may play a crucial role. In the absence of microbubbles, FUS would typically require higher energy levels to achieve a similar effect on the BBB. This could increase the risk of undesirable effects, such as thermal damage to the tissue or unwanted mechanical effects. These tiny, gas-filled bubbles oscillate when hit by ultrasound waves, creating pressure changes in the surrounding blood vessels. This action, known as cavitation, gently disrupts the tight junctions between endothelial cells in the BBB resulting in temporary openings that disrupt the blood brain barrier (23). 8 of the 10 studies which used microbubbles reported successful BBB disruption (8, 12–14, 16–19). The 2 studies that used microbubbles but did not report BBB opening theorized that this was likely due to subtherapeutic acoustic pressure being used in both studies (20, 21). Their theory was based on the threshold acoustic pressure to observe therapeutic BBB opening in previous animal studies (24). The 2 studies that did not use microbubbles did not specifically measure BBB disruption (9, 15). The results of the 12 included studies suggest the use of FUS with microbubbles for BBB opening is reversible and safe, with the BBB appearing to return to its normal state within 40 hours, which offers a promising method for treating neurological diseases by facilitating direct drug delivery to the brain. However, further research is essential to fully understand the optimal dosages, administration techniques, mechanisms of action, and safety profiles for this innovative therapeutic approach.

### Other therapeutic effects of FUS in AD

The 12 studies primarily focused on the feasibility and safety of BBB disruption. The mechanisms by which FUS might directly affect Alzheimer’s pathology, beyond facilitating drug delivery, needs further elucidation. For instance, whether FUS can induce a therapeutic effect by stimulating neuronal activity or promoting the clearance of pathological proteins remains an area for future research. Additionally, the absence of negative effects on cognitive measures in the 12 studies is encouraging, but the potential for FUS to improve cognitive outcomes in AD patients has not been conclusively demonstrated. 1 of the 12 studies reported a positive impact on certain cognitive tests following FUS treatment (20). Specifically, they reported improvements in immediate recall and recognition memory on the Seoul Verbal Learning Test after the sonication procedure. This suggests that FUS, beyond its potential to facilitate drug delivery through BBB disruption, might have direct beneficial effects on certain cognitive functions in Alzheimer’s disease patients. However, the variable follow-up periods and lack of standardized cognitive assessment in these studies underscores the need for well-designed clinical trials focusing on cognitive endpoints.

### Participant demographics

The geographical diversity of the studies underlines the universal appeal of FUS as a potential therapeutic strategy, yet it also calls for standardized protocols to better facilitate comparative analysis and the ability to replicate findings across different populations. The majority of the 12 studies participants mean age was greater than 65 and there were more females than males in most of the 12 studies which is reflective of the demographics of people most commonly diagnosed with AD. The participants’ race wasn’t explicitly stated in any of the 12 studies, therefore it’s not possible to assess if the geographical diversity of the studies translated into the participants of these 12 studies being racially diverse. The absence of younger participants with early-onset Alzheimer’s as well as the lack of information on the racial diversity of participants limits the generalizability of these findings. Future studies should aim for a more inclusive representation, particularly with regards to race, ethnicity, and age, in order to fully assess FUS’s efficacy and safety across the entire spectrum of AD patients (25, 26).

### Therapeutic applications of FUS beyond AD

There are several potential therapeutic applications of FUS mediated BBB opening beyond the treatment of AD. For example, as a potential method of enhancing the delivery of drugs to some brain tumors (27). Furthermore, at higher intensities FUS can be used, as a non-invasive method of thermal ablation, for the treatment of several neurological disorders such as Parkinson’s disease, Major Depressive Disorder, and Obsessive Compulsive Disorder (28). While FUS appears promising for the treatment of some neurological disorders, it is crucial to consider both the quality and quantity of available evidence, as well as the effectiveness of more established treatment modalities used for each specific disorder.

### Future directions

The exploration of FUS for BBB disruption in AD treatment is promising, potentially offering a novel avenue to enhance drug delivery to the brain. The safety profile and successful BBB opening observed provides a solid foundation for future research. However, the translation of these preliminary successes into tangible clinical benefits is hindered by the fact that the results of these studies are not generalizable. Additionally, it’s important to acknowledge that while the successful penetration of gadolinium-based contrast agents demonstrated in 8 of the 12 studies is promising it does not mean that therapeutic agents used to treat AD, for example Aducanumab, will achieve similar penetration at target sites through combination with FUS therapy; additional studies will be necessary to explore the penetration of these agents before clinical benefits might be achieved. An animal study published in 2022 demonstrated enhanced penetration of Aducanumab at target sites using FUS with microbubbles (5). The use of standardized methodology and equipment, control groups, broader participant demographics particularly regarding age and race, multi-institutional collaboration, and longer follow up periods would all help to improve the generalizability of future human studies on this topic. Furthermore, some future studies should look beyond the use of FUS as an adjunct treatment and explore its possible direct effects on AD in humans. Overall, future studies should include randomized controlled trials and try to elucidate important topics such as the long-term impact of FUS-mediated BBB disruption in AD patients, the impact of FUS on cognitive outcomes in AD patients, as well as the direct effects of FUS on AD pathology. In conclusion, while FUS mediated BBB opening shows promise as a potential therapeutic modality for AD, its relevance and utility for the treatment of the rapidly growing number of AD patients predicted worldwide (29) will depend on both the undertaking and results of gold standard trials on this topic.

## Data availability statement

The original contributions presented in the study are included in the article/Supplementary material, further inquiries can be directed to the corresponding author.

## Author contributions

KS: Conceptualization, Data Curation, Investigation, Formal Analysis, Methodology, Visualization, Writing – original draft, Writing – review & editing. SK: Investigation, Formal Analysis, Writing – review & editing.
