# Supplementary material for: Focused ultrasound therapy for Alzheimer’s disease: exploring the potential for targeted amyloid disaggregation
Source: Front Neurol. 2024 Aug 6;15:1426075. doi: 10.3389/fneur.2024.1426075 (PMC11333319; doi:10.3389/fneur.2024.1426075)
Supplement: Supplementary file 1 [file Table_1.DOCX]

| **Study** | **Study Design** | **Sample**  **Size** | **Age**  **(Years)**  **and**  **Sex** | **OCEBM Levels** | **Intervention** | **Outcome** | **Location** | **Funding** |
| --- | --- | --- | --- | --- | --- | --- | --- | --- |
| Mehta et al.  (2023) [12] | Phase II prospective clinical trial | 8 | Range: 54-76  Mean: 65  5 males and 3 females | 4 | Focussed transcranial ultrasound with systemic microbubbles (Definity)  Frequency: 220 kHz  Acoustic Power: Unspecified. Automatically modulated by the system's feedback control based on the detected acoustic activity.  Pulse Duration: 5 milliseconds.  Repetition Time: 1 second  Treatment Duration: Typically, 90 seconds for each treatment target (per session)  Timing: 3 sessions at 2-week intervals  Target Areas: Hippocampal, parietal, and frontal brain regions | Immediate BBB opening was confirmed by the extravasation of intravenously administered contrast agent at all targeted sites.  Rapid clearance of interstitial contrast agent as well as BBB closure within 24–48 h of FUS intervention at all treated sites of each participant.  No serious adverse event was encountered by any trial participant. | United States | Insightec |
| Meng et al.  (2023) [16] | Phase II prospective clinical trial | 9 | Range: 59-79  Mean: 70  4 males and 5 females | 4 | Focussed transcranial ultrasound with systemic microbubbles (Definity)  Frequency: 220 kHz  Acoustic Power: Unspecified. Automatically modulated by a cavitation feedback controller based on the acoustic activity during sonication.  Pulse Duration: Unspecified  Repetition Time: Unspecified  Treatment Duration: Ranged from 45-170 minutes, averaging 122 minutes (cumulative across all sessions)  Timing: 3 sessions at 2-week intervals  Target Areas: Bilateral precuneus, bilateral anterior cingulate cortex, and unilateral/bilateral hippocampi | Increased parenchymal contrast-enhancement on T1-weighted MRIs demonstrated successfully increased BBB permeability post-sonication within all targets.  One day post-treatment, resolution of the enhancement indicated restoration of the BBB permeability in all cases; however, 21 of the 139 (15%) regions still demonstrated hyperintensities in adjacent sulcal regions on contrast-enhanced FLAIR sequence.  Clinical examination did not show any new neurological deficits or serious AEs related to the procedure | Canada | Insightec |
| Jeong et al.  (2022) [20] | Preliminary clinical trial/ Pilot study | 8 | Mean: 78.1  Standard Deviation: 2.9  1 male and 7 females | 4 | Focussed transcranial ultrasound with systemic microbubbles (Definity)  Frequency: 250 kHz  Acoustic Power: Unspecified.  Pulse Duration: 20 milliseconds.  Repetition Frequency: 2 Hz  Treatment Duration: 180 seconds (per session)  Timing: 1 session.  Target Areas: Right hippocampus | Radiological evidence of contrast enhancement associated with BBB opening was found in neither the visual inspection nor the ICA of the DCE–MRI data.  The immediate recall and recognition memory on the Seoul Verbal Learning Test were significantly improved after the sonication.  No adverse events were observed during the hospitalization and follow-up outpatient visits for 5 to 24 months. | South Korea | Neurosona Co., Ltd., and Korea Health Industry Development Institute, and Korea Dementia Research Centre, and National Research Foundation of Korea. |
| Epelbaum et al.  (2022) [19] | Phase I/II clinical trial | 9 | Median: 71.0 [69.0, 73.0]  4 males and 5 females | 4 | Focussed ultrasound, using SonoCloud-1 implantable ultrasound device, with systemic microbubbles (SonoVue). The SonoCloud-1 was implanted at the left parietotemporal junction centred on the left supramarginal gyrus.  Frequency: 1 MHz  Acoustic Power: The acoustic pressure, initially set at 0.9 MPa, was increased after the first sonication session to 1.03 MPa.  Cycles Per Pulse: A 25,000-cycle pulse was used every second.  Treatment Duration: 4 minutes (per session)  Timing: Over 3.5 months, patients underwent seven ultrasound sessions, which were performed twice per month  Target Areas: Left supra-marginal gyrus. | Detectable ultrasound-mediated BBB disruption was observed for 10/16 (62.5%) of the sessions with available T1 maps.  One severe adverse event occurred during the trial. An Independent Data Safety Monitoring Board concluded there was an unlikely relation of this AE to the BBB opening procedure, and the participant agreed to continue sonication on the protocol.  No statistically significant change was observed on cognitive measures. | France | BPI France, and Carthera. |
| Rezai et al.  (2022) [13] | Open-label prospective clinical trial | 10 | Range: 55-76  3 males and 7 females | 4 | Focussed transcranial ultrasound with systemic microbubbles (Definity)  Frequency:  220 kHz  The ultra-sound power output varied between 4 W and 11.5 W during each 90-second sonication. The power selected was between 50% and 60% of the ramped power setting that induced subharmonic band acoustic spectra (cavitation halt).  Each sonication site received 2.6-msec pulses spaced by 30.4 msec for a total of 10 cycles. A 1550-msec rest period followed sonication of all four sites within a target volume. This cycle was then repeated for a total of 90 seconds.  Target Areas: Hippocampus and EC in the first 6 participants, expanded for increased volume and additional targeting of frontal and parietal lobes in the subsequently enrolled participants. | Immediate BBB opening of the FUS target region was demonstrated by parenchymal contrast enhancement in all participants and sessions. The BBB opening, which was determined by contrast enhancement, was confined to the FUS target region and did not occur in other brain regions.  All BBB openings were transient, and BBB closure occurred within 24–48 hours in all targeted brain regions of all participants.  Participants tolerated the procedure well, with no procedure-related serious AEs.  At the 6-month follow-up (n = 10), cognitive function was stable compared to baseline. At the 1-year follow-up (n = 7), cognition showed a decline in the overall group, similar to what was observed in the ADNI cohort. | United States | Insightec, and National Institute of General Medical Sciences of the NIH, and ADNI. |
| Mehta et al.  (2021) [8] | Prospective phase II clinical trial | 3 | Range: 61-73  Mean: 69  3 females | 4 | Focussed transcranial ultrasound with systemic microbubbles (Definity)  Frequency: 220-kHz  The ultra-sound power output varied between 4 W and 11.5 W during each 90-second sonication. The power selected was between 50% and 60% of the ramped power setting that induced subharmonic band acoustic spectra (cavitation halt).  Each sonication site received 2.6-msec pulses spaced by 30.4 msec for a total of 10 cycles. A 1550-msec rest period followed sonication of all four sites within a target volume. This cycle was then repeated for a total of 90 seconds.  Timing: 3 sessions at 2-week intervals  Target Areas: Hippocampus and entorhinal cortex | Contrast extravasation was identified within the treated brain volumes immediately after completion of all sonication treatments, indicating focal spatially precise BBB opening.  MRI repeated 24 hours later revealed resolution of parenchymal contrast enhancement at all treated sites, with return of baseline T1 signal intensity and no evidence of new parenchymal enhancement after repeated administration of gadolinium-based contrast agent.  Study participants remained stable at follow-up neurologic and neuropsychologic examinations, with no clinical deterioration after 19 months (16 months for participant 2, 12 months for participant 3) after completion of therapy. | United States | National Institute of General Medical Sciences of the National Institutes of Health, and National Institute of Neurological Disorders and Stroke. |
| Jeong et al.  (2020) [21] | Single centre prospective study | 4 | Mean: 78.8  Standard Deviation: 3.3  1 male and 3 females | 4 | Focussed transcranial ultrasound with systemic microbubbles (Definity)  Frequency: 250 kHz  A tone-burst-duration of 20 ms with a pulse repetition frequency of 2 Hz (i.e., a duty cycle of 4%). The acoustic intensity at the focal target had a spatial-peak pulse-average acoustic intensity of 0.5-3 W/cm2, resulting in a spatial-peak temporal-average acoustic intensity of 0.02-0.12 W/cm2.  Treatment Duration: 3 minutes  Timing: 1 session  Target Areas: Right hippocampus | The visual inspection and ICA analysis of the DCE-MRI data revealed no evidence of contrast enhancement related to BBB disruption.  No adverse events were observed after the procedure or in outpatient follow-up visits for up to 1 year.  Overall, mild improvement was observed in global cognitive function (MMSE), executive function (Contrasting Program, Go/No-Go Test), and memory (SLVT-E: immediate recall and recognition). | South Korea | Neurosona Co., Ltd., and KHIDI, and KDRC, and National Research Foundation of Korea, and Institute for Bio-Medical convergence, Incheon St. Mary’s Hospital. |
| Rezai et al.  (2020) [14] | Phase II clinical trial | 6 | Range: 55-73  Mean: 67  1 male and 5 females | 4 | Focussed transcranial ultrasound with systemic microbubbles (Definity)  Frequency: 220 kHz  Timing: 3 sessions at 2-week intervals  Target Areas: Hippocampus/EC (right and left variably) | MRI with gadobutrol IV contrast post-FUS treatment revealed immediate hippocampal parenchymal enhancement at the target region in all 17 treatment sessions, indicating enhanced BBB permeability, with no off-target enhancement.  This parenchymal contrast enhancement resolved within 24 h after FUS treatment.  There were no treatment-related adverse effects or neurological changes (up to 15 mo post-FUS). | United States | Insightec. |
| Beisteiner et al.  (2019) [9] | Pilot study | 35 | Unspecified | 4 | Focussed transcranial ultrasound.  Frequency: 2 MHz  Energy flux density: 0.2 mJ/mm².  Pulse Duration: 3 microseconds  Repetition Time: 200 milliseconds  Treatment Duration: Each pulse's sonication duration is 0.003 milliseconds (ms), with a total of 6000 pulses delivered across each session.  Timing: 3 sessions weekly for a duration of 2-4 weeks.  Target Areas:  For Center 1 (Austria) specific AD-relevant brain regions targeted included: Bilateral frontal cortex (dorsolateral prefrontal cortex and inferior frontal cortex extending to Broca's area)  Bilateral lateral parietal cortex (extending to Wernicke's area)  Extended precuneus cortex  Center 2 (Germany) used a non-navigated global brain stimulation approach without specifying particular target regions. | BBBO was not specifically measured.  At 3-months follow-up no adverse side effects were observed.  Following FUS treatment, significant enhancements in the cognitive states of patients were observed and maintained over a span of three months.  Upregulation of the memory network was detected in resting-state fMRI data after FUS therapy.  Increases in activation within the bilateral hippocampus after FUS therapy were verified by task-fMRI data. | Austria and Germany | Medical University of Vienna and the University of Vienna, and STORZ Medical. |
| Nicodemus et al.  (2019) [15] | Open-label pilot study | 22  AD: 11  PD: 11 | Range: 40 to 95  AD: 8 males and 3 females  PD: 8 males and 3 females | 4 | Focussed transcranial ultrasound.  Ultrasound was delivered using a 2 MHz transducer at a power of 520 mW/cm.  The study sought to facilitate deep sleep to minimize interference and ensure that the brain's natural processes could proceed with minimal external disruptions.  Treatment Duration: One hour  Timing: Eight consecutive sessions spaced one week apart  Target Areas: The mesial temporal lobe was targeted in AD and the substantia nigra was targeted in PD cases. | ASL imaging sequences performed immediately before and after one 1-hour session of ultrasound for a subsample of two patients showed markedly increased perfusion at the targeted hippocampal region after ultrasound relative perfusion. However, BBBO was not specifically measured.  No adverse events were reported.  Of all, 62.5% of patients had one or more improved cognitive scores without data incongruence, 87% had stable or improved fine motor scores, and 87.5% had stable or improved gross motor scores. | United States | This research did not receive any specific grant from funding agencies in the public, commercial, or not-for-profit sectors. |
| Meng et al.  (2019) [17] | Open label pilot study | 12  AD: 8  ALS: 4 | AD: mean age 67  PD: mean age 62  Sex Unspecified | 4 | Focussed transcranial ultrasound with systemic microbubbles (Definity)  Frequency: 220 kHz  Sonication volumes covered a rectangular spot approximately 9 mm by 9 mm, comprised of 3-by-3 grid of spots, each 3 mm in diameter. For the last three patients, given the extent of atrophy on their MRI, a 2-by-2 grid was utilized, yielding a spot approximately 5 mm by 5 mm. The device electronically steered the ultrasound through each grid for 50 s total, sonicating each spot with 2 ms on and 28 ms off bursts for 300 ms, with a repetition interval of 2.7 s (duty cycle 0.74%).  Timing: 2 sessions approximately 1 month apart  Target Areas: Pre-frontal lobe, Hippocampus, Anterior cingulate cortex, Posterior parietal cortex, Primary motor cortex | No adverse side effects were reported in any of the participants who underwent MRgFUS-induced BBB opening.  Enhanced permeability of the blood-brain barrier (BBB) to gadobutrol was observed across all targeted regions following sonication.  Contrast-enhanced FLAIR imaging revealed heightened signal intensity encircling numerous large cortical veins, such as the veins of Labbe and Trolard, extending into the subarachnoid space adjacent to the sonicated regions, indicating the persistence of glymphatic efflux post Focused Ultrasound-Microbubble (FUS-MB) induced BBB opening in human subjects. | Canada | Focused Ultrasound Foundation, and W. Garfield Weston Foundation, and ALS Society of Canada. |
| Lipsman et al.  (2018) [18] | Open label, prospective, proof-of-concept, phase I trial | 5 | Mean: 66.2  3 males and 2 females | 4 | Focussed transcranial ultrasound with systemic microbubbles (Definity)  Frequency: 220 kHz  Sonication volumes covered a rectangular spot approximately 9 mm by 9 mm, comprised of 3-by-3 grid of spots, each 3 mm in diameter. For the last three patients, given the extent of atrophy on their MRI, a 2-by-2 grid was utilized, yielding a spot approximately 5 mm by 5 mm. The device electronically steered the ultrasound through each grid for 50 s total, sonicating each spot with 2 ms on and 28 ms off bursts for 300 ms, with a repetition interval of 2.7 s (duty cycle 0.74%).  Timing: 2 sessions approximately 1 month apart  Target Areas: primarily targeted white matter in the frontal lobe, attempting to be specific to the dorsolateral prefrontal cortex where possible, given anatomic constraints. | Immediately after sonication, a discrete rectangular-shaped gadolinium enhancement can be seen in the targeted region on T1-weighted images.  At 24 h following the procedure, there was resolution of enhancement in the targeted region, indicating closure of the BBB.  No patient experienced a serious adverse event during this study. | Canada | Insightec, and Focused Ultrasound Foundation. |
